# Age and time effects on children’s lifestyle and overweight in Sweden

**DOI:** 10.1186/s12889-015-1635-3

**Published:** 2015-04-10

**Authors:** Lotta Moraeus, Lauren Lissner, Linda Olsson, Agneta Sjöberg

**Affiliations:** Department of Food and Nutrition, and Sport Science, University of Gothenburg, Box 300, Gothenburg, SE-405 30 Sweden; Department of Public Health and Community Medicine, Institute of Medicine, University of Gothenburg, Gothenburg, Sweden

**Keywords:** Children, Lifestyle, Longitudinal, Obesity, Secular trends, IOTF, WHO

## Abstract

**Background:**

High physical activity, low sedentary behavior and low consumption of sugar-sweetened beverages can be markers of a healthy lifestyle. We aim to observe longitudinal changes and secular trends in these lifestyle variables as well as in the prevalence of overweight and obesity in 7-to-9-year-old schoolchildren related to gender and socioeconomic position.

**Methods:**

Three cross-sectional surveys were carried out on schoolchildren in grades 1 and 2 (7-to-9-year-olds) in 2008 (n = 833), 2010 (n = 1085), and 2013 (n = 1135). Information on children’s level of physical activity, sedentary behavior, diet, and parent’s education level was collected through parental questionnaires. Children’s height and weight were also measured. Longitudinal measurements were carried out on a subsample (n = 678) which was included both in 2008 (7-to-9-year-olds) and 2010 (9-to-11-year-olds). BMI was used to classify children into overweight (including obese) and obese based on the International Obesity Task Force reference. Questionnaire reported maternal education level was used as a proxy for socioeconomic position (SEP).

**Results:**

Longitudinally, consumption of sugar-sweetened beverages ≥4 days/week increased from 7% to 16% in children with low SEP. Overall, sedentary behavior >4 hours/day doubled from 14% to 31% (p < 0.001) and sport participation ≥3 days/week increased from 17% to 37% (p < 0.001). No longitudinal changes in overweight or obesity were detected. In the repeated cross-sectional observations sedentary behavior increased (p = 0.001) both in high and low SEP groups, and overweight increased from 13.8% to 20.9% in girls (p < 0.05). Overall, children with high SEP were less-often overweight (p < 0.001) and more physically active (p < 0.001) than children with low SEP.

**Conclusions:**

Children’s lifestyles changed longitudinally in a relatively short period of two years. Secular trends were also observed, indicating that 7–9-year-olds could be susceptible to actions that promote a healthy lifestyle. Socioeconomic differences were consistent and even increasing when it came to sugar-sweetened beverage consumption. Decreasing the socioeconomic gap in weight status and related lifestyle variables should be prioritized. Primary school is an arena where most children could be reached and where their lifestyle could be influenced by health promoting activities.

## Background

Several lifestyle factors have been linked to overweight and obesity in children; among those are the consumption of soft drinks and sedentary behavior [1,2]. These factors often differ between children in areas and families characterized by varying socioeconomic circumstances [1,3]. Different socioeconomic markers have also been linked to childhood overweight and obesity in Sweden and other Nordic countries, where the prevalence is higher in children from families with low socioeconomic position (SEP) [4-6]. Parental education is often used as a proxy for child SEP [7] and in our previous study in a national sample of Swedish children aged 7 to 9 years, children with low maternal education were more often overweight and obese [1]. Other factors influencing child adiposity in our previous study was child sports participation ≥3 days/week and parental exercise which were inversely associated with overweight and obesity. Further, direct positive associations were observed with sedentary behavior >4 hours/day, having a computer in the bedroom and overweight [1].

Surveillance of lifestyle factors that relate to obesity is important in order to know how children’s habits change as they grow older or as a response to societal changes. This information is essential for planning of interventions and policy to be able determine which groups in the population would benefit most and which lifestyle factors should be targeted [8]. Since it is likely that differing social circumstances influence how an intervention is received, it is important to take such factors in to account also in surveillance [9]. Another important factor is gender of the child, which could influence lifestyle habits. In a Swedish survey including children aged 11, 13 and 15 years, boys more often participated in sports after school, but also had a less healthy diet in all age groups [10].

The aim of this study was to examine sports participation, sedentary behavior and some dietary factors as well as weight status (overweight and obesity) and how these develop longitudinally from grades 1–2 and over two years. Further, we investigate secular trends in the same variables measured in repeated cross-sectional samples in children in grades 1–2 in three measurements points over five years: 2008, 2010 and 2013. We also investigated whether the trends differ according to gender or socioeconomic position.

## Methods

The current survey uses regional data from a nationally representative sample of Swedish schoolchildren measured in 2008 [11], which was part of the World Health Organization (WHO) Childhood Obesity Surveillance Initiative (COSI) [12,13]. Lifestyle factors of children and their parents were collected using a family questionnaire, and children’s weights and heights were measured. Thirteen countries took part in the first data collection in 2008 [14]. Two more studies were conducted in 2010 and 2013 in which Sweden participated at a regional level.

**Figure 1:**
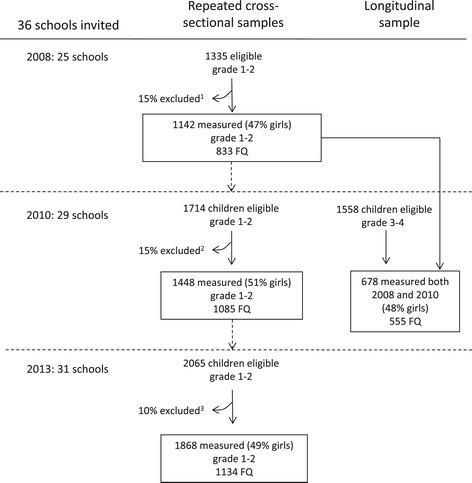
**Inclusion process of cross sectional surveys in 2008, 2010 and 2013 and longitudinal study in 2008 and 2010.** Number of children eligible, measured and with completed family questionnaires. ^1^Reasons for exclusion in 2008: 90 parent refusals, 82 sick/absent, 19 child refusals, 2 miscellaneous. ^2^Reasons for exclusion in grade 1–2 2010: 140 parent refusals, 85 sick/absent, 16 child refusal, 10 miscellaneous. ^3^Reasons for exclusion in 2013: 57 parent refusals, 119 sick/absent, 16 child refusals, 5 miscellaneous.

The study consisted of measurements of weight and height performed in schools together with questionnaires completed by parents about their own and their child’s background and lifestyle. The first survey was part of the national data collection where a national sample of 220 primary schools was randomly and proportionally selected from the National School Registry [1,11]. Sampling was based on type of municipality and whether the schools were public or independent. All schools were invited and 94 schools agreed to participate. Participating schools were considered to be representative based on the sampling criteria. The inclusion process and participation rates of the current regional study are illustrated in Figure 1. In 2008, 36 schools in West Sweden were invited to participate and 25 schools accepted the invitation. All schools were once again invited to participate in a follow-up study in 2010 and 2013, and 29 and 31 schools agreed, respectively. In 2008, 2010, and 2013, a total of 4358 children in grades 1 and 2 were measured. In 2010, grades 3 and 4 were also included in schools that had participated in 2008, and based on parental consent to follow children’s anthropometric measurements longitudinally, we were able to identify 678 children who were measured both in 2008 and 2010.

The standardized protocol used in the national study [1,11] was approved by the Regional Ethical Review Board in Stockholm in 2008 (No. 2008/309–31/5), while the Regional Ethical Review Board in Gothenburg advised that ethical approval would not be required (No. 070–08). However, the Regional Ethical Review Board in Gothenburg subsequently approved the protocol in 2013 (No. 761–12). Opt-out consent was used in all schools in 2008 and 2013 but in 2010 five schools chose to use active consent; this has been described in detail elsewhere [15]. Measurements were performed at school using portable equipment. Height was measured to the nearest 0.1 cm and weight to the nearest 0.1 kg. Children wore light clothing and no shoes during measurements.

### Family questionnaire

A family questionnaire (FQ) was distributed to the children by teachers on the day of measurement and brought home to be filled out by parents. The FQ was developed by the WHO and translated into Swedish. The questions were not validated but designed to be comparable across countries participating in the European COSI study. In 2008 and 2010, sealed envelopes containing the completed questionnaires were collected by the teachers and sent to the researchers. However, in 2013 parents were asked to mail the questionnaires directly to the researchers as requested by the ethical committee that year.

The FQ contained questions about children’s physical activity and sedentary behavior, diet, and parental background. Parents were asked how many hours per day (never, less than one hour, one hour, two hours, three hours or more) their child spent on reading/homework, watching TV and using the computer on weekdays and weekends. Approximate hours per day were calculated and the three variables were combined into approximate hours of sedentary behavior per day and dichotomized into ≤4 hours/day and >4 hours/day. Children’s sports participation was dichotomized into <3 days/week and ≥3 days/week. Parents reported children’s consumption of sugar-sweetened beverages (SSB) and fruits through a food frequency questionnaire. In 2008, 4 response categories were available: never, 1–3 days/week, 4–7 days/week or every day. After evaluation of the first data collection the decision was made to increase the categories to 8 in the following two surveys: never, 1–3 days/month, 1 day/week, 2–3 days/week, 4–6 days/week, once every day, twice every day, and 3 times a day or more [16]. The same categorization was used all years: consumption of SSB was dichotomized into ≤3 days/week or >3 days/week and fruits consumption was categorized as daily or not. These dichotomizations were used for practical reasons since more detailed categories were lacking.

Parents also reported their level of education and employment status. Maternal education ≤12 years or >12 years was chosen as a proxy for socioeconomic position (SEP). In longitudinal analyses, information from the baseline was used to define maternal education, which was included for 659 children (in 97 children, maternal education was collected from the second questionnaire in 2010). Employment status was reported as a) public employment, b) private employment, c) self-employed, d) student, e) homemaker, f) unemployed, g) retired, or h) other. Employment status was dichotomized as employed (categories a through c) and not employed (categories d through h).

### Calculations and statistics

BMI was calculated and classified into overweight and obesity according to the International Obesity Task Force (IOTF) [17] and the World Health Organization 2007 growth standard (WHO 2007) [18]. Overweight includes obesity in both references. To analyze secular trends in the prevalence of overweight, obesity, and lifestyle variables as well as differences between groups (gender, SEP), we performed generalized linear regression analyses using the GENLIN command in SPSS. The school code was included as a repeated subject in all models, to take multilevel sampling into account. Cross-sectional trends in lifestyle variables were adjusted for weight status as overweight/not overweight. The longitudinal data was also analyzed using the GENLIN command with school and child included as repeated subjects. Cohen’s Kappa was used to test the agreement between weight categories in 2008 and 2010. To test for effect modification by SEP and gender, interaction terms were included in both longitudinal and cross sectional analyses. Weight classification and lifestyle variables are presented with percentages. Statistical significance was established at a p-level of <0.05. Statistical analyses were performed with SPSS 22.0 (IBM Corp. Released 2013. IBM SPSS Statistics for Windows, Armonk, NY: IBM Corp.).

## Results

### Longitudinal sample

#### Dietary factors

**Figure 2:**
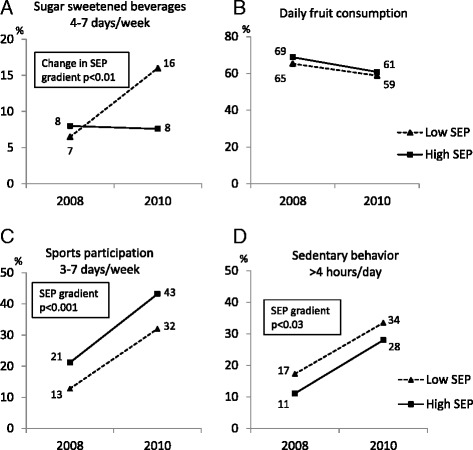
**Longitudinal changes in lifestyle according to maternal socioeconomic position (SEP).** Change in SEP gradient was observed in sugar sweetened beverages 4–7 days/week **(A)**. SEP gradients were observed in sports participation 3–7 days/week **(C)** and sedentary behavior >4 hours/day **(D)**. Daily fruit consumption was stable and no SEP gradient was observed **(B)**. P-values were calculated with generalized linear regression model including schools as repeated subject.

Of the 678 children who were measured in 2008 (age 7–9 years) as well as in 2010 (age 9–11 years), 555 children (82%) returned the FQ both years. Consumption of SSB 4–7 days/week increased overall from 7% at age 7–9 years to 12% at age 9–11 years (p = 0.006). A significant effect modification by SEP was observed (p = 0.01): consumption of SSB 4–7 days/week more than doubled from 7% to 16% in the low-SEP group while remaining stable in children with high SEP (Table 1, Figure 2A). Daily fruit consumption decreased overall (Figure 2B). Slightly more girls than boys consumed fruit daily: 69/67% of girls compared to 66/53% of boys in 2008/2010, (overall p = 0.004).

**Table 1:** **Longitudinal and cross sectional trends in sports participation, sedentary behavior, consumption of sugar-sweetened beverages (SSB) and fruit**

	**Longitudinal measurements**			**Cross sectional measurements**			
Measurement year	2008	2010		2008	2010	2013	
Max number of respondents (N)	N = 555	N = 555	Total trend p-value^1^	N = 833	N = 1085	N = 1134	Total trend p-value^2^
	%	%		%	%	%	
Sports 3–7 d/w	16.7	37.0	<0.001	23.3	24.8	26.7	0.28
Sedentary behavior >4 h/d	14.1	31.0	<0.001	15.1	19.6	22.2	0.001
SSB 4–7 d/w	7.1	11.9	0.006	8.8	10.2	6.5	0.04
Fruit daily	67.0	59.7	0.01	66.2	62.9	61.6	0.11

^1^Trends between 2008 and 2013, p-values were calculated with generalized linear regression model including schools as random intercept, adjusted for overweight.

^2^Difference between grades 1–2 in 2008 and grades 3–4 in 2010, p-values were calculated with generalized linear regression model including schools as repeated subject.

#### Sedentary behavior and sports

Percentage of children reporting participation in sports 3–7 days/week as well as sedentary behavior >4 hours/day approximately doubled from 17% and 14% respectively in the longitudinal sample (p for trends in both variables <0.001) (Table 1). There were consistent SEP gradients in both variables with higher levels of sedentary behavior and fewer days of sport participation per week in children with low SEP (Figures 2C, D). No gender differences were observed.

#### Weight status

The majority of the 678 children who were measured both years, 93% when applying IOTF and 87% when applying WHO 2007, remained in the same weight category; Cohen’s Kappa was 0.72 (IOTF) and 0.65 (WHO 2007). The prevalence of overweight and obesity was stable overall (Table 2). Prevalence of overweight by IOTF was about twice as high in children with low SEP at 18.9/19.1% in 2008/2010 compared to those with high SEP at 8.6/7.6% in 2008/2010. There were no gender differences either year.

**Table 2:** **Prevalence of overweight and obesity overall and according to gender, in the longitudinal sample** ^**1**^ **(n = 678) and cross sectional samples** ^**2**^ **(total n = 3052)**

	**Girls**			**Boys**			**Total**				
Measurement year	2008	2010	2013	2008	2010	2013	2008	2010	2013	Time x gender interaction p-value	Time x SEP interaction p-value
Weight classification	%	%	%	%	%	%	%	%	%		
Longitudinal sample (n)	(326)	(326)		(352)	(352)		(678)	(684)			
*Overweight*	12.0	11.7	-	15.3	15.1	-	13.7	13.4	-	0.96	0.47
*Obesity*	2.5	2.1	-	2.8	2.0	-	2.7	2.1	-	0.58	0.92
Cross-sectional sample (n)	(406)	(565)	(549)	(427)	(520)	(585)	(833)	(1085)	(1134)		
*Overweight*	13.8	19.5	20.9^3^	16.2	14.8	13.3	15.0	17.2	17.0	0.004	0.44
*Obesity*	2.7	2.8	2.9	2.6	1.9	1.4	2.6	2.4	2.1	0.23	0.27

^1^Difference between grades 1–2 in 2008 and grades 3–4 in 2010, p-values were calculated with generalized linear regression model including schools and child as repeated subject.

^2^Trends between 2008 and 2013, p-values were calculated with generalized linear regression model including schools as repeated subject.

^3^P-value for trend <0.05.

### Cross-sectional samples

#### Changes in participation and social characteristics

Participation of 7–9-year-old children with FQs was lower in 2013 compared to previous years. This was observed in both girls and boys. A significantly higher proportion of mothers, 56%, reported an education level >12 years in 2013 compared to 46/45% in 2008/2010, (p <0.001). No change in the distribution of employed mothers was observed: (p = 0.1) (Table 3).

**Table 3:** **Participation and characteristics of the cross-sectional samples**, **all children and children with family questionnaire (FQ)**

**Measurement year**	**2008**	**2010**	**2013**
Participation			
All measured children (n, % of eligible)	1142 (86)	1448 (85)	1868 (91)
*Girls/boys (% of eligible)*	85/86	84/85	91/90
Completed FQ (n, % of measured)	833 (73)	1085 (75)	1134 (61)
*Girls/boys (% of measured)*	76/70	76/74	61/61
Distribution of children according to individual socioeconomic characteristics from FQ			
*Maternal education % low/high*	54/46	55/45	44/56
*Maternal employment*	79/21	82/18	85/15
*% employed/not employed* ^*1*^			

^1^Without employment includes unemployed, retired, homemakers, students and miscellaneous.

#### Dietary factors

**Figure 3:**
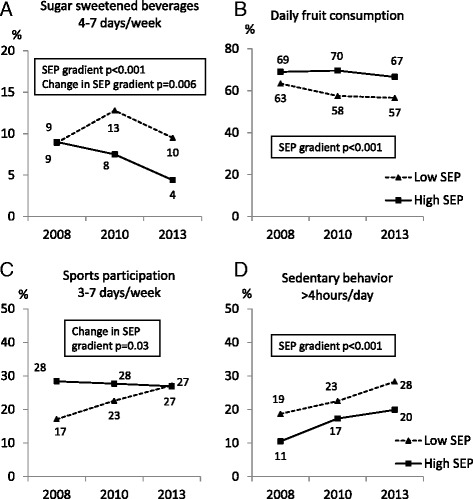
**Cross sectional trends in lifestyle according to maternal socioeconomic position (SEP).** SEP gradients were observed in consumption of sugar sweetened beverages 3–7 days/week **(A)**, daily fruit consumption **(B)** and sedentary behavior >4 hours/day **(D)**. Change in SEP gradient was observed in sugar sweetened beverages 4–7 days/week **(A)** and sports participation 4–7 days/week **(C)**. P-values were calculated with generalized linear regression model including schools as repeated subject.

Overall, consumption of SSB 4–7 days/week decreased in the repeated cross-sectional samples from 8.8% to 6.5% (p = 0.04), while daily fruit consumption remained stable (p = 0.11) (Table 1). The decrease in consumption of SSB was driven by decreasing consumption in the high-SEP group while no change was observed in the low-SEP group, resulting in a gradient between the groups (p for change in SEP-gradient = 0.006) (Figure 3A). The SEP gradient in daily fruit consumption was consistent (Figure 3B). Slightly more girls than boys consumed fruit daily: 67/67/64% of girls compared to 65/59/59% of boys in 2008/2010/2013, (overall p = 0.002).

#### Sedentary behavior and sports

About one in four 7–9-year old children participated in sports 3–7 days/week each year. In 2008, children with low SEP were reported to participate in sports fewer days per week than those in the high-SEP group: 17% compared to 28% reported sports participation 3–7 days/week (p = 0.001). This SEP gradient disappeared due to increased sports participation in the low-SEP group in the cross-sectional measurements in 2010 and 2013 (Table 1, Figure 3C). There was also a gender difference, where boys generally participated in sports 3–7 days/week more often than girls: 26/30/31% of boys compared to 21/20/22% of girls in 2008/2010/2013, (overall p = 0.002). Children engaging in sedentary behavior >4 hours/day increased significantly from 15% in 2008 to 22% in 2013. An overall consistent SEP gradient was observed with higher levels of inactivity in the low-SEP group (p = 0.03) (Figure 3D). The level of sedentary behavior was higher in boys: 19/30/32% compared to 23/24/27% in girls in 2008/2010/2013, (overall p = 0.01).

#### Weight status

The mean age was 8.4, 8.5, and 8.4 years in 2008, 2010, and 2013 respectively. Prevalence of overweight and obesity was stable overall, but the prevalence of overweight increased cross-sectionally from 14% to 21% in girls (p <0.05). There was an overall gender difference in both overweight (p <0.001) and obesity (p = 0.03) with higher prevalence in girls (Table 2). There were also consistent overall SEP gradients in overweight (p = 0.001) and obesity (p = 0.001). In 2008 19% of children in the low-SEP group and 11% in the high-SEP group were overweight. Corresponding numbers for obesity were 3.6% in the low-SEP group and 1.7% in the high-SEP group. Similar figures were observed in 2010 and 2013.

When applying the WHO 2007 reference to the cross-sectional measurements, the prevalence of overweight was 25.4%, 26.9% and 25.5% in 2008, 2010 and 2013, respectively (p for trend = 0.8). Corresponding figures for obesity were 7.8%, 7.6% and 7.5% (p for trend = 0.9). Overall SEP gradients were detected for overweight (p < 0.001) and obesity (p < 0.001). Contrary to when applying the IOTF reference, no increase in overweight in girls was observed, but similarly, obesity levels were higher in girls: 9% compared to 6% in boys (p < 0.001).

## Discussion

In this study of schoolchildren living in West Sweden, we were able to observe longitudinal effects over two years and cross-sectional trends over a five-year period. Consumption of SSB increased in children with low SEP in the longitudinal sample and decreased cross-sectionally in children with high SEP; this resulted in socioeconomic differences. Sedentary behavior increased both cross-sectionally and longitudinally, while sports participation increased only longitudinally.

### Age, time, and SEP aspects of lifestyle

In two recent review articles, consumption of SSB was linked to overweight and obesity in children [19,20]. Consumption of SSB has also been linked to difficulties in meeting dietary guidelines of several micronutrients [21]. Therefore, consumption of SSB could be a marker for unhealthy dietary habits. In our longitudinal sample, we observed a doubling of SSB consumption from ages 7–9 years to ages 9–11 years in the low SEP group. An American study including somewhat older children than in our study, observed that mainly boys increased their consumption of SSB over five years, even after adjusting for SEP [22]. The Swedish part of the WHO study Health Behaviour in School-aged Children (HBSC) also found that in older children than in the current study, boys more often than girls consumed SSB on a daily basis [10]. These differing results may reflect that gender plays a greater role in lifestyle choices among older children, while younger children are more influenced by the socioeconomic position and the lifestyle of their family. Children also gain more independence as they grow older and are able to purchase their own food to a larger extent.

In our cross-sectional samples, we found that SSB consumption decreased in children with high SEP and was stable in those with low SEP at ages 7 to 9 years. In a repeated cross-sectional study where Norwegian 11- and 12-year-olds answered a questionnaire about usual beverage habits, consumption of SSB decreased in children with both low and high parental education between 2001 and 2008 [23]. Studies from the United States have found that SSB consumption in children has decreased [24,25] or has been stable [26] during the last ten years. Similar to our study, others have found that consumption of SSB was more prevalent in low SEP groups [3,23,24]. An American study examining cross sectional trends in food and beverage consumption from the 1980s to 2010 found that SSB was a major contributor to energy intake in children aged 2–18 years [27]. There was an increase in energy intake and consumption of SSB from 1989 to about 2004, at which time a decline in both was observed. Although this study did not report trends in SSB consumption by SEP groups, it was concluded that the observed decrease in energy intake only occurred in high income families and not in low income families [27]. In that study, other top ten contributors to energy intake were foods such as pizza, snacks and deserts. This is similar to a Swedish national survey of children’s dietary habits [28]. In 2003, 25% of children’s energy intake came from energy dense foods with low nutritional value such as SSB. Considering that we observed an increased socioeconomic gradient in consumption of SSB both in cross sectional and longitudinal observations in West Sweden, it is important to monitor changes in subgroups and on a national level.

Due to the nature of our food frequency questionnaire we could not assess the amount of fruit and vegetable children consumed [16]. In 2008, the highest frequency available to choose from was once every day, which does not give us an estimate of habitual daily intake and is difficult to compare to the dietary recommendations for fruit and vegetable consumption, which in Sweden is 400–500 grams every day depending on age [29]. We did, however, find that around 60% of children ate fruit daily, with a declining longitudinal trend. Girls and children with high SEP had a higher consumption than boys and children with low SEP, which is consistent with findings in one review article [30] that also found that younger children usually have higher fruit consumption than older children. The Swedish HBSC also found that boys more seldom ate fruit on a daily basis than girls [10].

In children followed longitudinally, the number of days participating in sports increased in all children, and there was a consistent SEP difference with higher sports participation in the high-SEP group. This is consistent with findings in a Swedish study of longitudinal changes in lifestyle over two years [31]. That study included three cohorts in Stockholm County and observed increased sport participation in all age groups including children in grade 2 [31]. An Australian survey using accelerometers found that moderate and vigorous after-school physical activity decreased over five years in two cohorts of 5-to-6-year-olds and 10-to-12-year-olds [32]. The use of objective and more detailed measures of physical activity in that survey may explain the discrepancies with our survey. Furthermore, they also studied duration of physical activity while we only asked for number of days of sports participation, which does not give any information of duration or intensity [32]. In the cross-sectional sample, the SEP gradient in sports participation disappeared due to increased sports participation in the low SEP group. A qualitative study including schools from different socioeconomic areas in a Scottish city found that lack of easy access to sport facilities and costs were factors limiting sports participation in children in low-SES areas [33]. We could not access this kind of information about our study area, but it would be of interest to investigate whether such changes influenced the increase in sports participation in the low SEP group.

In our previous study on the nationally representative sample in 2008, we found that children’s overweight and obesity were associated with high levels of sedentary behavior, similar to other’s observations [1,34]. In the current study, in which the baseline data collection was part of the national survey, we observed an increase in sedentary behavior longitudinally as well as between cohorts, deriving mostly from a change in screen time (data not shown). If this increase continues into adolescence, there is a risk that it will lead to excess weight gain in the group with high screen use [35]. Since sedentary behavior was consistently higher in the low SEP group, this could in extension lead to further social differences not only in sedentary behavior but also in obesity.

### Trends in overweight and obesity

The prevalence of overweight and obesity was higher when applying the WHO 2007 reference compared to the IOTF reference. This is commonly reported and is due to differing cut off points in the two references [15,36]. Whether applying the WHO 2007 reference or IOTF reference, the children followed longitudinally remained in their weight class, indicating stability in the growth trajectory. Other short- and long-term surveys following children longitudinally have also reported a significant agreement between the weight classifications over time, which is in line with our findings [37,38]. In the repeated cross-sectional samples, prevalence of overweight and obesity was stable overall, but an increase in overweight in girls was observed when applying the IOTF reference but not the WHO 2007 reference. Both references for weight classification revealed stable SEP gradients in overweight and obesity consistent with findings in the national sample from 2008 [11]. Other studies, including our previous regional study on a similar sample of children, indicate that the social gradient in weight status may be increasing [15,39]. In our previous study, an area proxy for socioeconomic status was used [15]. Including questionnaire based information reduces the number of participants and contributes to a selected sample. However, similar socioeconomic gradients were observed whether we used the area classification or the individual SEP information as in the current study.

### Strengths and limitations

The baseline of the current study includes the regional part of WHO Childhood Obesity Surveillance Initiative. Even though we were not able to implement a continuous national surveillance system, we could perform two additional data collections in the large region of West Sweden. All data collections followed the same protocol as other participating COSI countries, including standardized measurements, trained staff, and common questionnaires. We were able to detect changes in weight classification and lifestyle both overall and when stratifying by SEP and gender. In the longitudinal sample, despite the relatively short time period, we seem to have captured a pivotal age when children’s lifestyles are susceptible to change and when SEP gradient may increase. Limiting the cross-sectional part of this study is the fact that we were forced to change the method for how the FQ was returned in the last study year. This could have contributed to the lower participation rate in 2013 and to the change in distribution of maternal education. Maternal employment rates were, however, stable. Further, results in the current study differ from results when analyzing overweight in the 23 schools in West Sweden that were included all three years [15]. As discussed earlier, this may reflect differing participation rates which in turn may bias the results. Decreasing participation rates are a growing problem in epidemiological research when using questionnaire-based information and avoiding this problem may prove difficult [40]. However, since we have anthropometric information on children without FQs, we can attempt to account for the differing prevalence. Thus, to avoid underestimating or overestimating the trends in lifestyle variables associated with weight status, we adjusted the analyses for overweight. Furthermore, the adjustment did not affect the results. An additional limitation was the different approach in the process of parental consent among the three years. However, sensitivity analyses showed that the data collected from children measured with active consent did not affect our results. Our questionnaire data on physical activity and sedentary behavior cannot be used to assess energy expenditure or duration and intensity of the activity. However, there are several methodological issues to consider when assessing physical activity. Objective measures gain more detailed information over a short period of time, whereas questionnaire-based methods have the ability to include a larger number of participants and assess habitual activity. Finally, the study is limited by the lack of validation of the FQ developed by the WHO. We did however, find similar SEP gradients in physical activity and consumption of SSB as others have [23], and in the previous national sample we observed association between sedentary behavior and overweight, similar to other studies [1,34].

## Conclusions

To conclude, several lifestyle variables changed, both longitudinally and cross-sectionally. We found that lifestyle changes mainly occurred in the longitudinal sample and where a secular trend also occurred, as in sedentary behavior, the longitudinal effect was still larger. This may not be surprising since longitudinal effects are a combination of increasing age interacting with societal changes, both which influence children’s habits. It is worrying that social gradients in sedentary behavior and consumption of SSB are consistent or even increasing. These changes may further increase the social inequalities related to obesity. Since these changes occurred within a short period of time, it should also be possible to reverse any unhealthy behavior within a few years, and perhaps actions should be focused on at-risk groups. The fact that the social gradient in sports participation disappeared indicates that the trends can be reversed. Ideally, measures should be taken before unhealthy habits have been established, and primary school seems to be a perfect arena for promoting a healthy lifestyle. In the school setting, there is also a possibility to reach children from all societal groups and a chance to bridge the social gap. Based on our findings and previous literature, increased consumption of fruit, decreased consumption of SSB and decreased sedentary behavior could be promoted to boys and children in low-SEP groups while girls could be encouraged to be more physically active.
